# The intelligent Traffic Management System for Emergency Medical Service Station Location and Allocation of Ambulances

**DOI:** 10.1155/2022/2340856

**Published:** 2022-07-07

**Authors:** Ezzatollah Asgharizadeh, Mahsa Kadivar, Mohammad Noroozi, Vahid Mottaghi, Hamed Mohammadi, Adel Pourghader Chobar

**Affiliations:** ^1^Department of Industrial Management, University of Tehran, Tehran, Iran; ^2^Department of Business Management, Payame Noor University, Varamin Branch, Tehran, Iran; ^3^Department of Industrial Engineering and Management Systems, Amirkabir University of Technology, Tehran, Iran; ^4^Department of IT Management, Qeshm Branch, Islamic Azad University, Qeshm, Iran; ^5^Department of Industrial Engineering, Faculty of Industrial and Mechanical Engineering, Qazvin Branch, Islamic Azad University, Qazvin, Iran

## Abstract

In the present study, the optimization of medical services considering the role of intelligent traffic management is of concern. In this regard, a two-objective mathematical model of a medical emergency system is assessed in order to determine the location of emergency stations and determine the required number of ambulances to be allocated to the station. The objective functions are the maximization of covering the emergency demands and minimization of total costs. Moreover, the use of an intelligent traffic management system to speed up the ambulance is addressed. In this regard, the proposed two-objective mathematical model has been formulated, and a robust counterpart formulation under uncertainty is applied. In the proposed method, the values of the objective function increase as the problem becomes wider and, with a slight difference in large dimensions, converge in terms of the solution. The numerical results indicate that, as the problem's complexity increases, the robust optimization method is still effective because, with the increasing complexity of the problem, it can still solve large-scale problems in a reasonable time. Moreover, the difference between the value of the objective function in the proposed method and the presence of uncertainty parameters is very small and, in large dimensions, is quite logical and negligible. The sensitivity analysis shows that, with increasing demand, both the number of ambulances required and the amount of objective function have increased significantly.

## 1. Introduction

Emergency medical services are an important part of the health system, an effective system that provides rapid response to calls, patient transfer, and timely treatment, ultimately, saving human lives, accountable to the prehospital health system [1]. Medical emergency organizations are divided into two main categories, affiliated to the United Kingdom and the United States and affiliated to France and Germany, including on-site treatment, and the accelerated response has not been significant [2].

On the other hand, in healthcare management systems, time is a significant and influential factor. In large cities, however, there are always significant delays in the arrival of patients' positions due to heavy traffic. For this reason, it is essential to design a traffic management system in order to provide required routes for health vehicles and minimize the travel time to the patient's position [1, 3].

Accordingly, this research is based on the use of uncertainty to respond to healthcare visiting demand as quickly as possible. In this regard, stochastic models target the inherent uncertainty and dynamics of the medical emergency system and are adapted to take into account the randomness of the initial contact process. Therefore, location-inspired models also consider demand-constrained costs and try to minimize the total cost of setting up stations and meeting demand.

To address demand uncertainty, first a definitive model and then a probabilistic model are presented, and it is assumed that each vehicle is capable of delivering a maximum certain number of emergency calls over the planning horizon. Moreover, like most coverage models, they need a vehicle that can meet emergency demand [4, 5]. The initial formulation is then expanded, and a two-step mathematical program is proposed to combine the uncertainties so that this is the first attempt to apply this method to an ambulance location, where two decision steps are considered. The first step is to determine the location of the standby locations and the second step is to assign the task of entering service requests to the standby locations. It should be noted that demand uncertainty manifests itself in the second stage. Next, an ambulance allocation model was proposed. In that model, minimizing the costs of operating and transporting the ambulance, as well as the requests that were not submitted on time, was addressed. To offer the best possible solution taking into account the diversity of demand, the newly introduced model provides a strong example of location-allocation formulation that aims to ensure that a solution to a set of demands can be solved. In a way, the concept shows the maximum number of simultaneous requests to estimate the number of vehicles to be stationed at each station.

In previous research, location optimization to meet emergency needs has been highly regarded. Goli and Malmir [6] have located and routed in crisis situations. In this research, the covering approach has been used. In this method, not all places with demand are serviced, and an attempt is made to cover the demand of the affected people in the nearest places. Fuzzy uncertainty has been used in this research. Pahlevan et al. [7] have examined the location and distribution of goods in conditions of uncertainty. In this research, gray wolf optimizer (GWO) and red deer algorithm (RDA) have been applied to solve the problem.

Zhang and Li [8] have proposed a mathematical model for locating emergency relief centers. In this research, fuzzy uncertainty and possibility planning have been used. Nickel et al. [9] have studied the problem of locating ambulance stations in the event of random demand. For this purpose, a sampling method is developed, and then, by optimizing a mathematical model, the best position of the stations is determined. Boujemaa et al. [10] have proposed a two-stage model for medical service in which service centers and service time are optimized.

After careful consideration of the previous research items, it is revealed that the uncertainty of demand has attracted the attention of researchers, and, according to their objectives and the field under study, the allocation of emergency vehicles has been considered in many different ways. However, it is challenging to compare their results at a common point. In addition, the robust formulation is still a considerable challenge given the computational effort required to solve them. One of the most useful data analysis tools is robust optimization suitable for decision-making under uncertainty. An intelligent traffic management system has also been used to accelerate the movement of vehicles and reach the patient as quickly as possible. In other words, the contribution of this research can be summarized as the following items:

- Integrating the home healthcare planning and intelligent traffic management
- considering two-stage programming to manage the intelligent traffic system and home healthcare routing
- considering uncertainty in demand parameters for home healthcare optimization and providing robust counterpart formulation to deal with such uncertainty

In this study, a robust formation is presented to formulate the problem with demand uncertainty. This formulation ensures with a certain probability that the number of vehicles stationed at a location can meet the maximum number of simultaneous demands. The sampling approach has been implemented to select the ambulance standby locations as well as the number of ambulances in each of the selected locations. In this case, it is assumed that demand follows a certain discrete distribution. Next, the proposed approach seeks to minimize fixed costs while ensuring the level of coverage for all scenarios considered. The results, based on a set of random cases, confirm the use of a random approach to deal with the problem under study. In a relatively similar work, a two-stage stochastic location model is proposed for the design of a robust two-stage medical emergency system, which simultaneously measures the location of ambulance stations, the number, and type of ambulances that must be deployed and determines the required areas of each station. Next, the average sample approximation algorithm is used to solve the problem.

Therefore, in this research, an attempt has been made to use more up-to-date methods in solving optimization problems and, in particular, robust optimization, which solves the problems caused by uncertainties in the input data and in the model parameters, which are discussed in Section 2. Next, the findings are analyzed in Section 3, and in Section 4, conclusions are provided.

## 2. Problem Description

**Figure 1:**
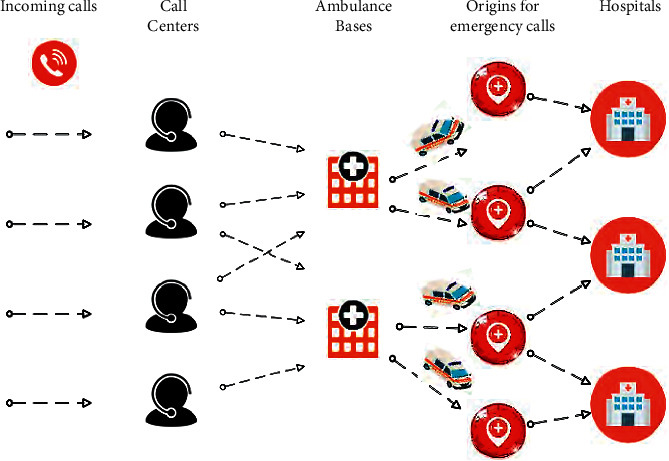
Overview of demand and allocation of ambulances to the demand place.

When an emergency call is made in a subarea, if there is one, the ambulance is transported from the station to the scene of the accident, and then the patient is transported to the hospital. Therefore, the sooner the patient is taken to the hospital, the sooner the treatment begins. In general, ambulances are located in preinstalled stations. When a call is made, the ambulance leaves the station at the scene and then returns to the station. The allocation of ambulances to the place of demand is shown in Figure 1.

Moreover, in this research, the intelligent traffic management system has been considered using a map. It predefines the ambulance route and the traffic light on the route and the status of the light (green or red) on the map. In this system, there is a control center that detects the current location of the ambulance and sends a command to the traffic light to turn the light green, or the light will continue to its current state. The goal is for the ambulance not to get in its way due to a red light.

The mechanism of this system is that it calculates the distance and direction of the ambulance, i.e., the distance between the ambulance and the next traffic light, and calculates the direction, i.e., determining the ambulance route at each intersection. Calculating the distance allows the control center to turn the traffic light green at the right time so that there is no traffic before the intersection, and calculating the route allows the control center to identify the traffic light at the next intersection. When the ambulance starts moving, its initial position is sent to the control center to determine the direction of movement and then to identify the nearest traffic light on the route. If the distance from the ambulance to the traffic light is less than a fixed value, the traffic light will show green; otherwise, the light will continue to its current state. It is the responsibility of radio systems to detect the movement of an ambulance, and a route is adopted that transports the ambulance to the scene of the accident or the place of request or the patient from the place of request to the hospital in the shortest possible time. After identifying this optimal route, the lights in the route and the distance of the ambulance to each intersection or lights are determined by the location and special radio systems to make arrangements at the desired time to minimize oncoming traffic.

The notations used in the research, including the sets, parameters, and decision variables, are expressed as follows.

### 2.1. Sets and Parameters

 
*I*: set of demand points
 
*J*: set of potential locations for stations
 
*L*: traffic light potential location set
 
*R*: set of ambulances that leave the station
 
*f*_*j*_: cost (daily) of building at station *j*
 
*p*_*j*_: cost of maintaining and purchasing each ambulance at station *j*
 
*d*_*ij*_: distance between the place of demand *i* and station *j*
 
*C*: cost per shipping unit
 
*μ*_*i*_: average demand (daily) at the place of demand *i*
 
*q*_*i*_: maximum simultaneous number of demands at the place of demand  *i*
 
*β*: standard distance for applying the intelligent traffic management system *l*
 
*H*_*l*_ : cost (daily) of each unit of the intelligent traffic management system *l*
 
*M*: a very large number
 
*W*: the weight of unmet demand
 
*N*_*l*_: the number of ambulances that, according to each demand, can be covered by the potential location of the intelligent traffic management system *l*

### 2.2. Decision Variables

 
*X*_*ij*_: percentage of demand at location *i* covered by station *j*
 
*Y*_*j*_: a binary variable and equal to 1 if the station is constructed at potential location *j*; otherwise, it is 0
 
*N*_*j*_: number of ambulances available at station *j*
 
*W*_*rl*_: a binary variable and equal to 1 when the distance from ambulance *r* to light *l* is greater than *β*; otherwise, it is 0
 
*Z*_*rl*_: a binary variable and equal to 1 when in light *l* for ambulance *r* turns green; 0 is the state when the light turns red
 
*S*_*l*_: a binary variable and equal to 1 when the traffic light *l* is in the potential location; otherwise, it is 0

### 2.3. Objective Function and Constraints

The certain model used to minimize the total cost, based on previous research [11, 12], is as follows:

(1) $$\begin{array}{c} P:\min\sum_{j\in J}f_{j}Y_{j}+\sum_{j\in j}P_{j}N_{j}+\sum_{j\in J}\sum_{i\in J}cd_{ij}\mu_{i}X_{ij}+\sum_{l}H_{l}S_{l}, \end{array}$$

(2) $$\begin{array}{c} \sum_{j\in J}^{s.t.}X_{ij}=1,\forall i\in I, \end{array}$$

(3) $$\begin{array}{c} X_{ij}\leq Y_{j},\forall i\in I,\forall j\in J, \end{array}$$

(4) $$\begin{array}{c} N_{j}\leq MY_{j},\forall j\in J, \end{array}$$

(5) $$\begin{array}{c} N_{l}\leq MS_{l},\forall l\in L, \end{array}$$

(6) $$\begin{array}{c} W_{rl}+Z_{rl}=1,\forall l\in L,\forall r\in R, \end{array}$$

(7) $$\begin{array}{c} \sum_{i\in I}q_{i\;}X_{ij}\leq N_{j},\forall j\in J, \end{array}$$

(8) $$\begin{array}{c} 0\leq X_{ij}\leq 1,\forall i\in I,\forall j\in J, \end{array}$$

(9) $$\begin{array}{c} Y_{j}\in \left\{0,\;1\right\},Z_{rl}\in \left\{0,\;1\right\},W_{rl}\in \left\{0,\;1\right\},S_{l}\in \left\{0,\;1\right\},\forall j\in J,\forall l\in L,\forall r\in R, \end{array}$$

(10) $$\begin{array}{c} N_{j}\in Z^{+},N_{l}\in Z^{+}\forall j\in J,\forall l\in L. \end{array}$$

The objective function in (1) includes minimizing the cost of construction of medical emergency system stations, the cost of emergency transportation equipment, the average cost of transportation between medical emergency system stations and the place of demand, and, finally, the cost of setting up and equipping an intelligent traffic management system. Constraint (2) guarantees the processing of each demand. Constraints (3) and (4) indicate the allocation of demand and transportation equipment only to open stations. Constraint (5) indicates the allocation of the intelligent traffic management system to the lights. Constraint (6) states how the intelligent traffic system operates when the distance from ambulance *r* to lamp *l* is less than the defined value. Constraint (7) states that the number of ambulances at station *j* should not be less than the maximum simultaneous demand. Constraint (8) represents the *Xij* range, and constraints (9) and (10) also represent the binary nonnegative variables.

In order to estimate the response rate of the medical emergency system, a maximum coverage model has been used, which ensures that the requests answered by EMS stations have a transfer time less than *T* [13, 14]. This concept is equivalent to minimizing the demands that are not covered at the time [15, 16]. Therefore, the following model is introduced to minimize fixed costs, equipment costs and purchases, and the cost of fines for applications that were not covered in the previous model.

(11) $$\begin{array}{c} O:\min\sum_{j\in J}f_{j}Y_{j}+\sum_{j\in j}P_{j}N_{j}+W\sum_{j\in J}\sum_{i\in I_{j}}\mu_{i}X_{ij}+\sum_{l}H_{l}S_{l},\\ \text{s}.\text{t}.\;\\ \text{Constraint}\left(2\right)∼\text{Constraint}\left(10\right). \end{array}$$

Here, *W* indicates the weight of applications that were not completed within the specified time. Moreover, in this objective function, *I*_*j*_ is defined as *I*/{*i* : *d*_*ij*_ ≤ *T* × *V*} where *V* is the average ambulance speed. By combining the two-objective functions (1) and (11), a definite two-objective model is created to design the problem of the medical emergency system, which will be used to compare with the robust model.

### 2.4. Robust Counterpart Formulation

In most cases of designing a medical emergency system, sufficient research data is not available to achieve a possible distribution function of parameters that include uncertainty. Therefore, developing a robust peer-to-peer approach is the most likely solution. In this research, the definite model used has become its robust counterpart by replacing the constraints that include the definite parameter with the constraints that include the set of uncertainties. That is, the uncertainty in the two parameters of the number of requests and the maximum simultaneous demand is considered. Moreover, the set of elliptical uncertainties along with safe parameters has been used to describe the uncertainties in this research.

In order to implement a robust counterpart model, first by inspiring the foundation of robust optimization, especially in the research of Ben-Tal et al. [17] and Goodarzian et al. [18], a standard formulation is created to convert the model to a robust counterpart form. The research is then applied to the mathematical model. Moreover, in order to solve the robust model, the parameters related to the robust counterpart form are quantified, and then the mathematical model is optimized.

### 2.5. Robust Formulation of Model *P*

To formulate a robust counterpart of the Model *P*, it is assumed that the number of emergency calls belongs to a set of elliptical uncertainties, which is shown in

(12) $$\begin{array}{c} U=\left\{U\in {\mathbb{R}}^{\left|I\right|}:U=\bar{U}+\Delta \varsigma ,\varsigma \leq \theta ,\bar{U}\in {\mathbb{R}}^{\left|I\right|},\Delta \in {\mathbb{R}}^{\left|I\right|\times \left|I\right|},\theta \in \mathbb{R}\right\}, \end{array}$$

where *U* is the vector of uncertain demand at places of demand and its numerical values are

(13) $$\begin{array}{c} U={\left[\mu_{1},\mu_{2},\ldots ,\mu_{n}\right]}^{T},\\ n=\left|I\right|,\\ \bar{U}={\left[{\bar{\mu }}_{1},{\bar{\mu }}_{2},\ldots ,{\bar{\mu }}_{n}\right]}^{T}. \end{array}$$

The matrix △=∑ ^1/2^  can be obtained through a separation method. Σ is actually the covariance matrix of emergency calls. *θ* is the safe parameter that is determined by derivatives and is also selected by the decision-maker to show the appropriate reaction to his approach to risk.

Moreover, the following constraints are considered for introducing the auxiliary variable *t*_*j*_.

(14) $$\begin{array}{c} t_{j}\geq \sum_{i\in I}d_{ij\;}\mu_{i}X_{ij},\forall j\in J. \end{array}$$

Accordingly, the robust counterpart approach can be obtained through the following model:

(15) $$\begin{array}{c} \underset{\mu \in U}{\max}\sum_{i\in I}d_{ij}\mu_{i}X_{ij}, \end{array}$$

(16) $$\begin{array}{c} \underset{\varsigma \leq \theta }{\max}{\left(\bar{U}+\Delta \varsigma \right)}^{T}X_{j}\leq t_{j}, \end{array}$$

where *X*_*j*_=[*d*_1*j*_*X*_1*j*_+*d*_2*j*_*X*_2*j*_+…+*d*_*nj*_*X*_*nj*_]^*T*^, *n*=|*I*|. Therefore, based on [15], it can be concluded that the robust counterpart of Constraint (7) can be reformulated as

(17) $$\begin{array}{c} {\bar{U}}^{T}X_{j}+\theta \sqrt{X_{j}^{\;T}\sum X_{j}}\leq t_{j}\forall j\in J. \end{array}$$

This means hedging against the standard distribution *θ*, provided that the coefficients belong to a set of elliptical uncertainties. The MNCD-based cone uncertainty set will be

(18) $$\begin{array}{c} \mathbb{Q}=\left\{Q\in {\mathbb{R}}^{\left|I\right|}:Q=\bar{Q}+\Xi \xi ,\xi \leq \beta ,\bar{Q}\in {\mathbb{Q}}^{\left|I\right|},\Xi \in {\mathbb{R}}^{\left|I\right|\times \left|I\right|},\beta \in \mathbb{R}\right\}, \end{array}$$

where numerical values are $\bar{𝒬}=\left[{\bar{q}}_{1},{\bar{q}}_{2},\ldots ,{\bar{q}}_{n}\right]\;\text{ and }𝒬=\left[q_{1},q_{2},\ldots ,q_{n}\right]$. Moreover, Ψ is the covariance matrix, and *β* is the safe parameter. Therefore, the robust counterpart of Constraint (7) will be as formulated as

(19) $$\begin{array}{c} {\bar{Q}}^{T}X_{j}+\beta \sqrt{X_{j}^{\;T}\Psi X_{j}\leq N}. \end{array}$$

As a result, the robust counterpart of the Model *P* will be as

(20) $$\begin{array}{c} P:\text{min}\sum_{j\in J}f_{j}Y_{j}+\sum_{j\in j}P_{j}N_{j}+\sum_{j\in J}ct_{j}+\sum_{l}H_{l}S_{l}, \end{array}$$

(21) $$\begin{array}{c} \sum_{i\in I}^{s.t.}d_{ij}{\bar{\mu }}_{i}X_{ij}+\theta \sqrt{\underset{i\in I}{\sum }\sigma_{i}d_{ij}^{\;2}X_{ij}^{\;2}}\leq t_{j},\forall j\in J, \end{array}$$

(22) $$\begin{array}{c} \;\underset{i\in I}{\sum }{\bar{q}}_{i}\;X_{ij}+\beta \sqrt{\sum_{i\in I}\Psi_{ij}^{\;2}X_{ij}^{\;2}}\leq N_{j},\forall j\in J, \end{array}$$

(23) $$\begin{array}{c} X_{ij}\in R_{+}\forall i\in I,j\in J. \end{array}$$

### 2.6. Robust Formulation of Model *O*

The robust formulation of Model *O* is formulated in a similar way to Model *P*. The third part in the objective function (11) is related to the uncertainty in the number of emergency calls that belong to the elliptical set, which is shown in

(24) $$\begin{array}{c} V_{j}=\left\{V\in {\mathbb{R}}^{\left|I_{j}\right|}:V=\bar{V}+\Lambda \varsigma ,\varsigma \leq \gamma ,\bar{V}\in {\mathbb{R}}^{\left|I_{j}\right|},\Lambda \in {\mathbb{R}}^{I_{j}\times I_{j}},\gamma \in \mathbb{R}\right\}, \end{array}$$

where *V* is the uncertain emergency demand vector generated by the following demand location *V*, *i* ∈ *I*_*j*_, *V*=[*μ*_*i*_1__, *μ*_*i*_2__,…,*μ*_*i*_*m*__]^*T*^, *m*=|*I*_*j*_|. Moreover, Λ=*ϕ*^1/2^ · *ϕ* is the covariance matrix; *μ* and *γ* are safe parameters. Similar to the method used in Constraint (14), we formulate a robust constraint counterpart as

(25) $$\begin{array}{c} {\tilde{t}}_{j}\geq \sum_{i\in I_{j}}\mu_{i}X_{ij},\forall j\in J, \end{array}$$

(26) $$\begin{array}{c} {\bar{V}}^{T}X_{j}+\gamma \sqrt{X_{j}^{\;T}\phi X_{j}}\leq {\tilde{t}}_{j},\forall j\in J. \end{array}$$

Therefore, the robust counterpart of Model *O* can be formulated as

(27) $$\begin{array}{c} O:\min\sum_{j\in J}f_{j}Y_{j}+\sum_{j\in j}P_{j}N_{j}+W\sum_{j\in J}{\tilde{t}}_{j}+\sum_{l}H_{l}S_{l}, \end{array}$$

(28) $$\begin{array}{c} \sum_{j\in J}^{s.t,}X_{ij}=1,\forall i\in I, \end{array}$$

(29) $$\begin{array}{c} X_{ij}\leq Y_{j},\forall i\in I,\forall j\in J, \end{array}$$

(30) $$\begin{array}{c} N_{l}\leq MS_{l},\forall l\in L, \end{array}$$

(31) $$\begin{array}{c} W_{rl}+Z_{rl}=1,\forall l\in L,\forall r\in R, \end{array}$$

(32) $$\begin{array}{c} \underset{i\in I}{\sum }{\bar{q}}_{i}X_{ij}+\beta \sqrt{\underset{i\in I}{\sum }\Psi_{i}X_{ij}^{\;2}}\leq N_{j},\forall j\in J, \end{array}$$

(33) $$\begin{array}{c} \underset{i\in I}{\sum }\mu_{i}X_{ij}+\gamma \sqrt{\underset{i\in I_{j}}{\sum }\sigma_{i}X_{ij}^{\;2}}\leq {\tilde{t}}_{j},\forall j\in J, \end{array}$$

(34) $$\begin{array}{c} 0\leq X_{ij}\leq 1,\forall i\in I,\forall j\in J, \end{array}$$

(35) $$\begin{array}{c} Y_{j}\in \left\{0,\;1\right\},Z_{rl}\in \left\{0,\;1\right\},W_{rl}\in \left\{0,\;1\right\},S_{l}\in \left\{0,\;1\right\},s \end{array}$$

Finally, a summary of the medical emergency system problem formulas is provided by considering the intelligent traffic management system as a robust two-objective optimization problem and is presented in

(36) $$\begin{array}{c} \min\sum_{j\in J}f_{j}Y_{j}+\sum_{j\in j}P_{j}N_{j}+\sum_{j\in J}ct_{j}+\sum_{l}H_{l}S_{l}, \end{array}$$

(37) $$\begin{array}{c} \min\sum_{j\in J}f_{j}Y_{j}+\sum_{j\in j}P_{j}N_{j}+W\sum_{j\in J}{\tilde{t}}_{j}+\sum_{l}H_{l}S_{l}, \end{array}$$

(38) $$\begin{array}{c} \sum_{j\in J}^{s.t.}X_{ij}=1,\forall i\in I, \end{array}$$

(39) $$\begin{array}{c} X_{ij}\leq Y_{j},\forall i\in I,\forall j\in J, \end{array}$$

(40) $$\begin{array}{c} N_{l}\leq MS_{l},\forall l\in L, \end{array}$$

(41) $$\begin{array}{c} W_{rl}+Z_{rl}=1,\forall l\in L,\forall r\in R, \end{array}$$

(42) $$\begin{array}{c} \underset{i\in I}{\sum }d_{ij\;}{\bar{\mu }}_{i}X_{ij}+\theta \sqrt{\underset{i\in I}{\sum }\sigma_{i}d_{ij}^{\;2}X_{ij}^{\;2}}\leq t_{j},\forall j\in J, \end{array}$$

(43) $$\begin{array}{c} \underset{i\in I}{\sum }{\bar{q}}_{i}X_{ij}+\beta \sqrt{\underset{i\in I}{\sum }\Psi_{i}X_{ij}^{2}}\leq N_{j},\forall j\in J, \end{array}$$

(44) $$\begin{array}{c} \underset{i\in I}{\sum }\mu_{i}X_{ij}+\gamma \sqrt{\underset{i\in I_{j}}{\sum }\sigma_{i}X_{ij}^{2}}\leq {\tilde{t}}_{j},\forall j\in J, \end{array}$$

(45) $$\begin{array}{c} 0\leq X_{ij}\leq 1,\forall i\in I,\forall j\in J, \end{array}$$

(46) $$\begin{array}{c} 0\leq X_{ij}\leq 1,\forall i\in I,\forall j\in J, \end{array}$$

(47) $$\begin{array}{c} N_{j}\in Z_{+},\forall j\in J, \end{array}$$

(48) $$\begin{array}{c} t_{j},{\tilde{t}}_{j}\in R_{+},\forall j\in Js, \end{array}$$

## 3. Numerical Results

In this section, in line with the previous sections and the process of achieving the research results, numerical tests are used to model the medical emergency system by considering the intelligent traffic management system. In this series of tests, the location of the emergency service stations and the facilities that are actually located in them are determined from the candidate stations for medical emergency services. Then, the required number of ambulances is determined based on the share of demand and its amount in each station. The data used in this section is based on the study of Ndiaye and Alfares [19], in which the location of the stations is proportional to the demand, which is dependent on the conditions in summer and winter. In order to better understand, the data used for solving the models are described in the following subsection.

### 3.1. Test Problem Design

The proposed model is an integer quadratic problem that can be solved through the bifurcation and delimitation algorithm to obtain the optimal solution [20–23]. Both certain and robust models have been solved using GAMS software and Windows 10 using a laptop that is equipped with an Intel Core i7 processor 8 GB of RAM. First, the performance of the proposed model is examined by comparing the two-objective functions that have been stated, namely, cost and effect functions.

A small case study has been conducted considering three emergency stations and five places of demand. The operating cost of allocating vehicles at stations *j* = 1, 2, 3 is 11,000,000, 56,000, and 130,000 units, respectively. The cost of each shipping unit is 50. The maximum simultaneous demand for the places of demand *i* = 1, 2, 3, 4, 5 is considered: 90, 19, 39, 183, and 103, respectively. The cost of the uncovered application is 50. The cost of station equipment for stations *j* = 1, 2, 3 is 115,700, 125,000, and 270,000 units, respectively. In the elliptical case, the values of $\bar{\mu }$ and $\bar{q}$ are presented in Tables 1 and 2.

In order to solve the model, both cases of the box and robust elliptical counterparts are optimized separately. Moreover, for the binary variables of integer, the bifurcation and delimitation method is applied. The model shows that the main station is constructed at the potential location *j* = 2 and the difference between the models is in the number of ambulances allocated. In the robust counterpart formulation, the model also operates based on quadratic cone optimization. In the box and elliptic cases, the model of the main problem goes out of the semi-infinite state, and the robust state must ensure the existence of an optimal global solution in the quadratic linear and conical states [17]. In addition, because the research problem involves a constraint that all applications must be covered, all requests are covered by station *j* = 2. The total number of vehicles required at the station *j* = 2 in all three definite, box, and elliptical cases are 434, 478, and 367, respectively. Moreover, the percentage of demand that is covered in location *i* by station *j* in all three definite, box, and elliptic modes is *X*_1,2_=*X*_2,2_=*X*_3,2_=*X*_4,2_=*X*_5,2_=1. In the following subsection, the effect of each parameter on the objective functions is examined separately.

### 3.2. Comparing the Objective Functions

Pareto solutions can be obtained by using the weighting method [20] and by assigning a weight commensurate with the objective functions. By combining the two-objective functions (1) and (11), it can be stated that

(49) $$\begin{array}{c} \min\sum_{j\in J}f_{j}Y_{j}+\sum_{j\in j}P_{j}N_{j}+\sum_{j\in J}ct_{j}+W\sum_{j\in J}{\tilde{t}}_{j}+\sum_{l}H_{l}S_{l}. \end{array}$$

**Figure 2:**
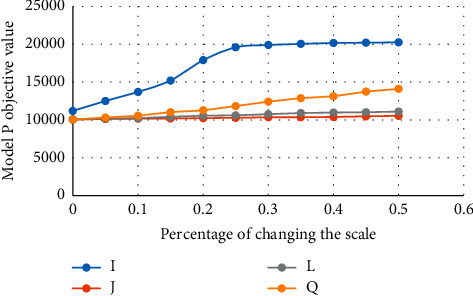
The effect of increasing the scale of the problem on the objective function of Model *P*.

The performance evaluation of the proposed model is done by assigning different weights as well as the different number of candidate stations. The number of candidate stations for the medical emergency system is 30, 50, and 70, respectively. As mentioned earlier, all data used in this study are taken previously. Moreover, in cases where explicit data were not available, ten experiments were performed in which the parameters were obtained by multiplying the numbers obtained from the previous data in an interval (1 + *ε*) where *ε* was obtained from a uniform distribution [−0.1, 0.1]. For more information, see [3, 5, 21–23]. The average speed of a car is 30 km/h. The average for each emergency job is one hour. The weight of uncovered demand is estimated at 50. The daily cost of the building at station *j*, i.e., *f*_*j*_, is 50 units, and the cost of purchasing and maintaining each ambulance unit at station *j*, i.e., *p*_*j*_, is two units. *T* also takes 15 minutes. The cost of each transport unit is estimated at 50. The maximum number of applications in the demand places is 10 units. The standard distance for applying the intelligent traffic management system is 200 units, and the cost of each unit of the traffic management system is 1 unit. According to the same demand that is considered for all three cases *j* = 30, 50, and 70, it is observed that the total number of ambulances required in different stations and in all three cases is 500 units and also increasing the number of stations from 30 up to 70 leads to an increase in costs, which can be fully seen in Table 3 and Figure 2.

Moreover, if an increasing trend can be found in the demand parameter, both the number of required ambulances and the value of objective function have increased significantly. By increasing the number of traffic light locations to apply the intelligent traffic management system, the objective function is first increased and then decreased. This may be due to the fact that as the number of intersections increases and, as a result, the traffic lights increase, the time for the ambulance to arrive from the station to the scene of the accident or from the place of demand to the hospital also increases and naturally leads to reduced response quality. Due to the lack of sufficient capacity in urban and intercity routes, costs increase. It is possible to increase the number of lights until there is a suitable platform for the application of an intelligent traffic management system, and therefore, in the absence of this, the system is not able to respond to all requests in a timely manner.

**Figure 3:**
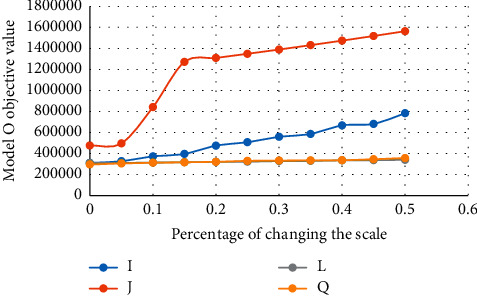
The effect of increasing the scale of the problem on the objective function of Model *O*.

Finally, the overall cost of the system is reduced. Moreover, as the number of simultaneous demands increases, in theory, system costs and the number of ambulances required increase dramatically to meet overall demand. It should be noted that the impact of other parameters on the objective function, such as the daily cost of the emergency station, the daily cost of emergency vehicles, and the daily cost of an intelligent traffic management system, as well as vehicle speed, will increase the objective function, which, due to this increase, leads to no significant changes made to the objective function; this is not used in the following analysis. The set of changes mentioned is illustrated in Table 4 and Figure 3.

**Figure 4:**
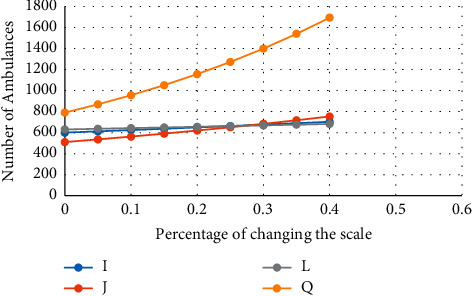
The effect of increasing the scale of the problem on the number of ambulances.

Moreover, the graph of the effect of the parameters on the number of ambulances required in both objective functions is as follows, in which the number of ambulances required changes with the change of the desired parameters, but, in the case of increasing the number of demand places, this change is more intense. And, in a way, it can be said that more ambulances are needed, which is possible by increasing the cost or increasing the objective function.  Figure 4 shows the changes in the number of ambulances and their effect on both objective functions.

## 4. Conclusion and Future Works

In designing a two-objective medical emergency system, considering the intelligent traffic management system, the parameters that include the demand are uncertain. To guide this important matter, an approach called robust optimization has been used. Despite the overly conservative solution in the box uncertainty mode, both this mode and the elliptical uncertainty have been used. The robust counterpart and its function were introduced, and the robust counterpart approach was converted to linear programming and quadratic programming. This means that the robust counterpart provides an optimal global solution.

Nowadays, home care programs have increased in response to the needs of patients at the community level; one of the reasons for this rapid growth is the proof of the efficiency of home care for patients in the face of different needs of patients, which has been proven by increasing the efficiency and effectiveness of this method. A study of 600 patients showed that 81% of patients referred for hospitalization could be successfully treated at home. In addition, hospital stays were reduced from approximately 12 days to 7 days, and only 12% of patients in home care needed to be hospitalized again. The widespread use of research tools in the field of healthcare in today's world indicates the effectiveness of these tools. There are several benefits to serving patients at home today. According to the given definition of patient visit activities, it is possible to provide medical services and patient care and health care at home.

By optimizing the provision of home health services, patients can benefit from such manufacturing services. These services can include a wide range of activities, such as a doctor's patient visit, delivery of medicine and medical equipment to the patient, and receiving laboratory samples and medicines. Moreover, equipment has not been used to provide maintenance services for medical equipment and devices at home. In countries where we see more resistance to infectious diseases due to the high consumption of antibiotics, the patient can develop more resistance to infectious diseases by going to the hospital. On the other hand, the cost of providing medical services at home is much lower than the high costs of hospitalization and the country's healthcare system.

It shows that the best way to provide home care is not to prevent nosocomial infections. Other benefits include a significant reduction in healthcare costs, a reduction in the number of intermediaries and timely and efficient service, and the avoidance of wasting time. Reducing the traffic load of the city and more patient comfort in such activities should not be underestimated.

In order to indicate the future outlooks, it is suggested to apply a multifactor robust optimization approach to deal with the uncertainty. Moreover, it can also include studies that consider risk management in medical emergency systems. Furthermore, some novel solution methods like gray wolf optimizer and red dear algorithm can be used. On the other hand, in the case of the intelligent traffic management system, some new technologies like the Internet of Things (IoT) can be considered in the proposed mathematical model.

## Figures and Tables

**Table 1:** The values of $\bar{\mu }$.

*i*	${\bar{\mu }}_{i}^{l}$	${\bar{\mu }}_{i}^{u}$	${\bar{\mu }}_{i}$
1	336	510	470
2	337	337	337
3	336	530	470
4	337	510	480
5	337	530	490

**Table 2:** The values of $\bar{q}$.

*i*	${\bar{q}}_{i}^{l}$	${\bar{q}}_{i}^{u}$	${\bar{q}}_{i}$
1	19	90	54.5
2	19	19	19
3	19	29	39
4	19	183	101
5	19	103	61

**Table 3:** The results of Model *P*.

	I	J	L	Q
0	11200	10050	10050	10050
5%	12500	10121	10190	10326
10%	13700	10163	10240	10562
15%	15200	10187	10412	11027
20%	17900	10239	10559	11276
25%	19600	10282	10612	11829
30%	19900	10355	10758	12414
35%	20050	10361	10908	12875
40%	20170	10396	10974	13141
45%	20200	10480	11023	13730
50%	20250	10546	11101	14107

**Table 4:** The results of Model *O*.

	I	J	L	Q
0	310200	475900	310300	295000
5%	327155	496635	310608	305520
10%	372825	840904	314181	312284
15%	395995	1271308	316860	316612
20%	475070	1309447	320803	321355
25%	507881	1348731	322527	330476
30%	558436	1389192	328203	333022
35%	585908	1430868	329706	335099
40%	667941	1473794	335171	335858
45%	681792	1518008	336705	345647
50%	782767	1563548	339967	357569

## Data Availability

The data used are included in the article. Codes are also available upon request.

## References

[1] Bélanger V., Ruiz A., Soriano P. (2019). Recent optimization models and trends in location, relocation, and dispatching of emergency medical vehicles. *European Journal of Operational Research*.

[2] Dick W. F. (2003). Anglo-American vs. Franco-German emergency medical services system. *Prehospital and Disaster Medicine*.

[3] Sajadi S. J., Ahmadi A. (2022). An integrated optimization model and metaheuristics for assortment planning, shelf space allocation, and inventory management of perishable products: a real application. *PLoS One*.

[4] Goodarzian F., Taleizadeh A. A., Ghasemi P., Abraham A. (2021). An integrated sustainable medical supply chain network during COVID-19. *Engineering Applications of Artificial Intelligence*.

[5] Ghasemi P., Khalili H. A., Chobar A. P., Safavi S., Hejri F. M. (2022). A New Multiechelon Mathematical Modeling for Pre-and Post disaster Blood Supply Chain: Robust Optimization Approach. *Discrete Dynamics in Nature and Society*.

[6] Goli A., Malmir B. (2020). A covering tour approach for disaster relief locating and routing with fuzzy demand. *International Journal of Intelligent Transportation Systems Research*.

[7] Pahlevan S. M., Hosseini S. M. S., Goli A. (2021). Sustainable supply chain network design using products’ life cycle in the aluminum industry. *Environmental Science and Pollution Research*.

[8] Zhang Z. H., Li K. (2015). A novel probabilistic formulation for locating and sizing emergency medical service stations. *Annals of Operations Research*.

[9] Nickel S., Reuter-Oppermann M., Saldanha-da-Gama F. (2016). Ambulance location under stochastic demand: a sampling approach. *Operations Research for Health Care*.

[10] Boujemaa R., Jebali A., Hammami S., Ruiz A., Bouchriha H. (2017). A stochastic approach for designing two-tiered emergency medical service systems. *Flexible Services and Manufacturing Journal*.

[11] Revelle C. (1989). Review, extension and prediction in emergency service siting models. *European Journal of Operational Research*.

[12] Jia H., Ordóñez F., Dessouky M. (2007). A modeling framework for facility location of medical services for large-scale emergencies. *IIE Transactions*.

[13] Araz C., Selim H., Ozkarahan I. (2007). A fuzzy multi-objective covering-based vehicle location model for emergency services. *Computers & Operations Research*.

[14] Goldberg J. (2004). Operations research models for the deployment of emergency services vehicles. *EMS Management Journal*.

[15] Daskin M. (1997). Network and discrete location: models, algorithms and applications. *Journal of the Operational Research Society*.

[16] Shen Z.-J. M., Daskin M. S. (2005). Trade-offs between customer service and cost in integrated supply chain design. *Manufacturing & Service Operations Management*.

[17] Ben-Tal A., El Ghaoui L., Nemirovski A. (2009). *Robust Optimization*.

[18] Goodarzian F., Hosseini-Nasab H., Muñuzuri J., Fakhrzad M.-B. (2020). A multi-objective pharmaceutical supply chain network based on a robust fuzzy model: a comparison of meta-heuristics. *Applied Soft Computing*.

[19] Ndiaye M., Alfares H. (2008). Modeling health care facility location for moving population groups. *Computers & Operations Research*.

[20] Cohon J. (2004). *Multiobjective Programming and Planning*.

[21] Gaspar A., Oliva D., Hinojosa S., Aranguren I., Zaldivar D. (2022). An optimized Kernel extreme learning machine for the classification of the autism spectrum disorder by using gaze tracking images. *Applied Soft Computing*.

[22] Ganguly S., Bhowal P., Oliva D., Sarkar R. (2022). BLeafNet: a Bonferroni mean operator based fusion of CNN models for plant identification using leaf image classification. *Ecological Informatics*.

[23] Pourghader Chobar A., Adibi M. A., Kazemi A. (2021). A novel multi-objective model for hub location problem considering dynamic demand and environmental issues. *Journal of Industrial Engineering and Management Studies*.

